# Evaluating the efficacy of different praziquantel treatment regimens using egg and circulating anodic antigen (CAA) detection methods in a *Schistosoma mansoni* endemic area in northeastern Brazil

**DOI:** 10.1016/j.ijpddr.2025.100628

**Published:** 2025-11-26

**Authors:** Rosangela Lima de Freitas Galvão, Pytsje T. Hoekstra, Paul L.A.M. Corstjens, Marta Cristhiany Cunha Pinheiro, Angela Maria da Silva, Luciene Barbosa, Sidney Lourdes César Souza Sá, Govert J. van Dam, Fernando Schemelzer de Moraes Bezerra

**Affiliations:** a Federal University of Ceará, Faculty of Medicine, Postgraduate Program in Pathology, Rua Monsenhor Furtado, s/n - Rodolfo Teófilo, CEP 60441-750, Fortaleza, Ceará, Brazil; b Federal University of Ceará, Department of Clinical and Toxicological Analysis, Research Laboratory in Parasitology and Mollusc Biology, Rua Pastor Samuel Munguba, 1210 - Rodolfo Teófilo, CEP: 60430-372, Fortaleza, Ceará, Brazil; c Leiden University Center for Infectious Diseases (LUCID), Leiden University Medical Center, Albinusdreef 2, 2333 ZA, Leiden, the Netherlands; d Department of Cell and Chemical Biology, Leiden University Medical Center, Einthovenweg 20, 2333 ZC, Leiden, the Netherlands; e Federal University of Sergipe, Department of Medicine and Postgraduate Program in Health Sciences, Rua Cláudio Batista S/N, Sanatório, CEP: 49.060-100, Aracaju, Sergipe, Brazil; f Federal University of Sergipe, Department of Morphology, Laboratory of Parasitology and Tropical Entomology, Av. Marcelo Deda Chagas, s/n - Bairro Rosa Elze, CEP 49107-230, São Cristóvão, Sergipe, Brazil; g State Government of Sergipe, State Health Department, Department of Epidemiological Surveillance, Av. Augusto Franco 3150, p – Ponto Novo, CEP 49097-670, Aracaju, Sergipe Brazil; h Federal University of Ceará, Faculty of Medicine, Postgraduate Program in Medical Sciences, Rua Prof. Costa Mendes, 1608 - 4° andar, CEP 60430-140, Fortaleza, Ceará, Brazil

**Keywords:** Efficacy, Safety, Praziquantel, Schistosomiasis, *Schistosoma mansoni*, Brazil

## Abstract

This study evaluated the efficacy of different treatment regimens with PZQ against *Schistosoma mansoni* infection. Residents of the Patioba and Colônia Miranda villages (Sergipe, Brazil) with a confirmed diagnosis of *S. mansoni* infection were randomized into one of the study groups and treated with a standard dose of PZQ (Group 1); with two doses spaced 24 hours apart (Group 2); or with two doses spaced 30 days apart (Group 3). Efficacy was assessed 30 days after the final treatment, based on the detection of eggs in feces using the Kato-Katz (KK) method and on the detection of CAA in urine using the Up-Converting Particle Lateral Flow (UCP-LF) assay. A total of 88 participants, who tested positive for infection by KK and UCP-LF CAA at baseline, were included in the statistical analysis. Cure rates (CRs) reached 100 % in all three study groups, according to KK. The UCP-LF CAA revealed that not all participants were cured, with the highest cure rate observed in Group 2 (69.6 %%), followed by Group 3 (57.7 %) and Group 1 (43.8 %). An Intensity Reduction Rate (IRR) of 100 % and >97 % was observed in all groups, based on KK and UCP-LF CAA, respectively. Reduction in *S. mansoni* burden was observed over time, with the IRR in all groups exceeding the efficacy threshold established by the WHO (>90 %). Cure rates varied according to the diagnostic method used, being overestimated when based on egg quantification, highlighting the importance of using more sensitive tools for the detection of active infections and monitoring the efficacy of PZQ.

## Highlights

• Two PZQ doses given 24 h apart achieved the highest antigenic CR.
• CRs were significantly lower when assessed with sensitive CAA detection.
• The UCP-LF CAA test proved more accurate than Kato-Katz for evaluating PZQ efficacy.
• All regimens reduced infection intensity beyond WHO efficacy threshold.

## Graphical abstract

**Figure:**
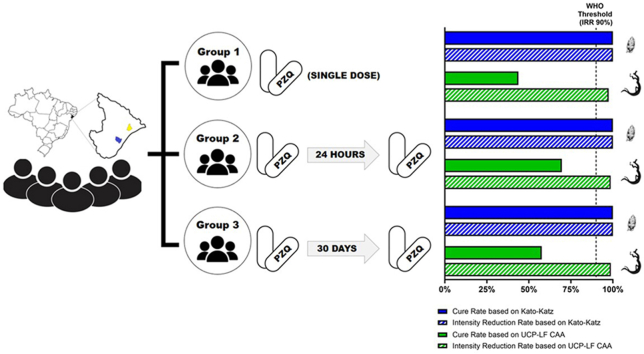

## 1 Introduction

Schistosomiasis is an important infectious parasitic disease of endemic nature, prevalent in 78 countries distributed across the African, Asian and South American continents (WHO, 2023). *Schistosoma mansoni*, the only species transmitting intestinal schistosomiasis in the Americas, is responsible for high chronic morbidity in endemic areas (Colley et al., 2014; WHO, 2023). In these areas, preventive chemotherapy with praziquantel (PZQ) is the main strategy for controlling morbidity caused by *S. mansoni*, with a recommendation from the World Health Organization for a single dose of 40 mg/kg of body weight (WHO, 2022). In Brazil, the treatment protocol recommended by the Ministry of Health is 60 mg/kg for children up to 15 years old and 50 mg/kg for adults, in a single dose (Brasil. Ministério da Saúde, 2024). Despite effectively reducing the parasite load, administering a single dose appears to be insufficient to eliminate the disease (Sabah et al., 1985; Doenhoff et al., 2008; Ross et al., 2015; Colley et al., 2017; McManus et al., 2018; Summers et al., 2022; Hoekstra et al., 2022a). Furthermore, immature forms of *Schistosoma* do not respond to treatment with PZQ (Doenhoff et al., 2008; Ross et al., 2015; McManus et al., 2018; Summers et al., 2022). Thus, the best approach for PZQ treatment as a tool to control schistosomiasis in endemic areas is still debated (King et al., 2011).

Studies on the efficacy of different PZQ treatment regimens in endemic areas of different countries report variable efficacy in terms of cure rate (CR) and Egg Reduction Rate (ERR) (King et al., 2011; Kabuyaya et al., 2018; Fukushige et al., 2021). Efficacy assessments measured by egg detection in high-risk communities in Africa indicate that after administration of PZQ in a single dose of 40–60 mg/kg for the treatment of *S. mansoni* infection, the CR ranged from 42 % to 79 % and in double doses between 69 % and 91 %. (King et al., 2011). In a recent review, treatment dose showed a positive impact on CR and ERR (Fukushige et al., 2021). Studies carried out in Brazil report a CR varying between 71.3 % and 79.2 % based on a single dose of PZQ (40–65 mg/kg) (Katz and Rocha, 1982; Carvalho et al., 1984; da Silva et al., 1986). A repeated-dose regimen seemed to increase the CR up to 90 % (da Cunha et al., 1987), whereas increasing the dose of PZQ did not improve the CR (de Queiroz et al., 2010; Olliaro et al., 2011). In general, prevalence as well as infection reduction estimates should be interpreted with caution, as they may be influenced by various factors, including the parasite's stage, sex, and pairing status (Nogueira et al., 2022), the host's age (Stothard et al., 2013), and the *Schistosoma* species (Fukushige et al., 2021). Other influencing factors include the PZQ dose and the time interval between doses in multiple-dose treatments, the post-treatment follow-up duration, the number of parasitological samples analyzed, and the diagnostic method used to assess the impact of drug treatment (Coulibaly et al., 2013; Sousa et al., 2019; Mazigo et al., 2021).

Most studies have used conventional microscopy for evaluating the efficacy of PZQ (Fukushige et al., 2021). Diagnostic tools for determining active *Schistosoma* infection based on the detection of circulating cathodic and circulating anodic antigen (CCA and CAA, respectively) have shown promise in overcoming the challenges of diagnosing schistosomiasis (Corstjens et al., 2020; Hoekstra et al., 2021; Ally et al., 2024). In different contexts of endemicity, both CCA and CAA have presented more accurate cure estimates when compared to cure estimates based on egg detection, since the quantity of *Schistosoma* eggs is subject to significant variability, due to egg fluctuation and the insensitivity of coproscopic methods, which results in false negatives results (Kongs et al., 2001; Berhe et al., 2004; Utzinger et al., 2015; Bezerra et al., 2021; Alemu and Nibret, 2024). PZQ efficacy assessed using UCP-LF CAA quantifies CAA in urine or serum samples. CAA is considered as a biomarker for active infection, as it is a glycoconjugate regurgitated by live *Schistosoma* worms into the host's circulation (Corstjens et al., 2020) and it has been demonstrated to be cleared within a few days to weeks after PZQ treatment (Sousa et al., 2019; Hoekstra et al., 2022a, Hoekstra et al., 2022b). The UCP-LF CAA test has demonstrated high sensitivity and specificity for detecting all *Schistosoma* species (Corstjens et al., 2020; Sousa et al., 2019; Knopp et al., 2015).

Studies carried out in the African territory have shown that the CR measured by antigen detection was significantly lower than that measured by Kato-Katz (KK), indicating that the efficacy of PZQ based on egg detection is overestimated (Coulibaly et al., 2013; Hoekstra et al., 2020; Mazigo et al., 2021; Hoekstra et al., 2022a). In fact, a study carried out in a low endemicity area in Brazil showed that the detection of CCA and CAA was a more sensitive approach for determining active *Schistosoma* infections as well as for the assessment of post-treatment cure (Sousa et al., 2019).

In the latest guideline for the control and elimination of human schistosomiasis published by the WHO, the importance of developing treatment strategies as well as conducting studies focused on the treatment of the disease aimed at monitoring PZQ efficacy in school and community settings is emphasized (WHO, 2022). Studies investigating the efficacy of different PZQ treatment regimens are mostly carried out in African countries and mainly focus on school-age children (Fukushige et al., 2021), while this type of research is scarce in the South American scenario. In South America, 95 % of the disease burden is concentrated in Brazil, where the disease is considered a serious public health problem (Katz, 2018). Studies investigating the efficacy of different treatment regimens with PZQ using more sensitive and specific diagnostic tools alongside or instead of conventional diagnostic methods, are of paramount importance and can contribute to the goal of eliminating schistosomiasis outlined by the World Health Organization (WHO, 2013a; WHO, 2022). The aim of the present study was to evaluate the efficacy of different PZQ regimens in combating *S. mansoni* infection in an endemic area of the state of Sergipe, Northeastern Brazil using traditional stool microscopy (KK) as well as more accurate CAA detection.

## 2 Material and methods

### 2.1 Ethical precepts

This study was part of a larger project entitled "Epidemiological, clinical-laboratory characterization and evaluation of therapeutic regimens of schistosomiasis mansoni in an area of high endemicity in northeastern Brazil". The research was carried out considering principles defined in Resolution 466/2012 of the National Health Council of the Ministry of Health/Brazil, with approval from the National Research Ethics Commission - CONEP under opinion number 6,590,838 and from the Research Ethics Committee (CEP) of the Federal University of Sergipe, Brazil, under opinion number 6,044,220.

All participants were informed about the purpose of this study and consent to voluntary participation in the study was obtained by signing the Free and Informed Consent Form (FICF). The participation of children and minors was consented to by their parents and/or guardians.

### 2.2 Study design, area and participants

This prospective and interventional study, carried out from August 2022 to November 2023, was conducted in two villages in the federative unit of Sergipe (SE), a state belonging to the Northeast Region of Brazil: Patioba village and Colônia Miranda village. The Patioba village is a quilombola community inhabited by 632 residents (IBGE - Instituto Brasileiro de Geografia e Estatística, 2022), located in a rural area of the municipality of Japaratuba. The Colônia Miranda village is a fishing community inhabited by 622 residents (SISAB - Sistema de Informação em Saúde para a Atenção Básica, 2023), located in the municipality of São Cristóvão.

#### 2.2.1 Inclusion criteria

In order to be eligible to participate in this study, individuals had to meet the following criteria.

- provide informed consent;
- have a confirmed *S. mansoni* infection based on Kato-Katz;
- aged ≥4 years;
- resident in Patioba village or Colonia Miranda village;
- have not received PZQ treatment in the last 3 months;
- be able and willing to provide multiple blood, urine and stool samples during the study.

#### 2.2.2 Exclusion criteria

Individuals who did not meet the inclusion criteria or who met any of the following criteria were excluded from participation in this study:

- Women who were menstruating, pregnant or suspected of being pregnant.

### 2.3 Schistosomiasis treatment and adverse reaction monitoring

After parasitological screening, eligible participants with a confirmed diagnosis of infection based on the Kato-Katz method (KK) were divided into three groups (1, 2 and 3) to receive praziquantel treatment. To reduce imbalances between groups regarding sex, age, and infection intensity, all diagnosed participants were initially listed in a Microsoft Excel® spreadsheet, stratified by parasite load, and subsequently ordered by age and sex. Based on this listing, participants were then alternately distributed into three new lists, with entries interleaved until the complete formation of the groups. Given the need for prior scheduling for intervention administration, there was no blinding of participants or teams to treatment. The three different treatment regimens were:

● Group 1: standard dose of PZQ;
● Group 2: standard dose of PZQ and after 24 h another standard dose of PZQ;
● Group 3: standard dose of PZQ and after 30 days another standard dose of PZQ.

PZQ was administered as recommended by the Brazilian Ministry of Health (Brasil. Ministério da Saúde, 2024): children (≤15 years) received a dose of 60 mg/kg of body weight (oral, single dose) and adults a dose of 50 mg/kg of body weight (oral, single dose). The place where the treatment was carried out was the residents' association of each village.

All participants received a standardized snack (fruit juice, coffee, bread, and biscuits) prior to administration of the PZQ dose and were duly supervised by a healthcare professional, pharmacist and a physician, to ensure that all took the full dose.

Participants were monitored for 24 h after each dose of PZQ. At the time of treatment administration, each individual received a leaflet containing space for adverse event recording and a dedicated telephone number for reporting adverse events. Active follow-up through telephone calls and home visits was conducted by the research team to ensure data collection from participants who did not spontaneously report adverse events. Those who experienced vomiting within the first hour after PZQ administration received a new dose of the medication. During this time, the infectious disease physician associated with the research group was available to evaluate and treat symptomatic events, when appropriate.

**Fig. 1:**
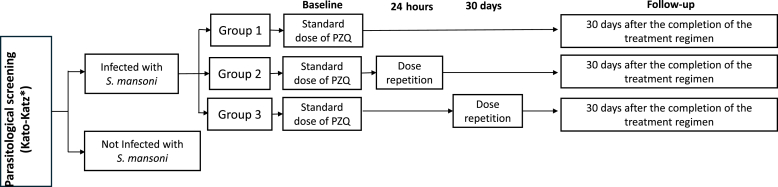
Flowchart showing the different groups and the different treatment regimens with praziquantel from the study.

A detailed description of the formation of the study groups is shown in Fig. 1.

### 2.4 Sample collection and processing

At baseline, two stool samples were collected from all study participants on consecutive days. Follow-up samples (1 stool sample and 1 urine sample) were collected 30 (D30) and 60 (D60) days after baseline treatment.

All urine samples were aliquoted and kept frozen at −80 °C in the Laboratory of Parasitology and Mollusc Biology of the Federal University of Ceará in Brazil, and subsequently sent to the Department of Parasitology, Leiden University Medical Center (LUMC), the Netherlands, for retrospective circulating antigen detection.

### 2.5 Diagnostic procedures

From each stool sample, two slides were prepared and analyzed as described by Katz et al. (1972).

Retrospectively, urine samples were tested at LUMC for the presence of CAA. Quantitative analysis of CAA was performed on urine samples using the up-converting reporter particle, lateral flow (UCP-LF) based assay, according to the protocol described elsewhere (Corstjens et al., 2020; Hoekstra et al., 2022a). A set of reference samples with a known CAA concentration was included to validate the cut-off (UCAA*hT*417 wet assay: 0.6 pg/ml) as well as to quantify individual CAA-levels (Corstjens et al., 2020).

### 2.6 Outcomes

The primary outcomes of the study was defined as parasitological and antigenic cure rate (CR) assessed 30 days after completion of the treatment regimen. The CR was defined as the proportion of participants being egg/CAA positive at baseline and who became egg/CAA negative 30 days after the last treatment (i.e. D30 for Group 1 and Group 2 and D60 for Group 3). Secondary outcomes included: egg reduction rates (ERR) and CAA intensity reduction rates (IRR), evaluated 30 days after the last treatment as well as CR, ERR and IRR 60 days after the last treatment for Group 1 and Group 2; and the safety of the (repeated) PZQ treatment regimens.

### 2.7 Statistical analysis

Only participants with a positive baseline outcome for both KK and UCP-LF CAA were included in the analysis. Data were summarized using descriptive statistics. The number and percentage positive for *Schistosoma* infection was determined for each diagnostic method. In case of KK, the intensity of *S. mansoni* infection was stratified in eggs per gram (EPG), and classified as light (1–99 EPG), moderate (100–399 EPG), or high (>400 EPG). Efficacy estimates were calculated based on WHO guidelines (WHO, 2013b). The CR was estimated based on the proportion of individuals positive at baseline and who became negative after the last treatment. The ERR or Intensity Reduction Rate (IRR) was estimated based on the intensity of infection after the last treatment compared to the intensity of infection before treatment. Figures were generated using GraphPad Prism for Windows (Version 10.2.3).

## 3 Results

**Fig. 2:**
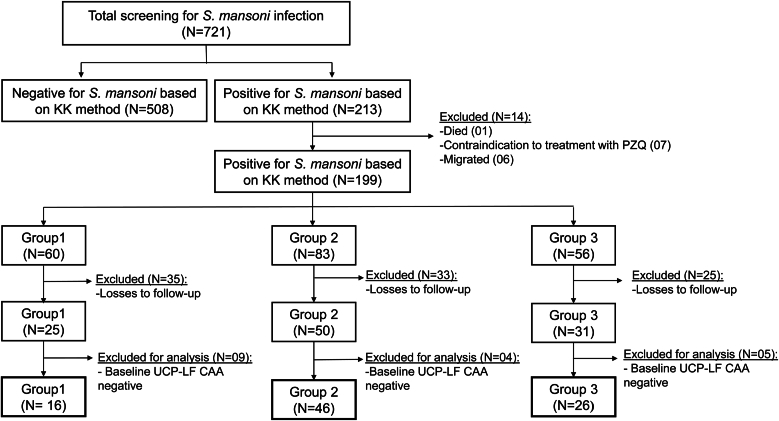
Flowchart showing screening, eligible participants for the study, and formation of treatment groups.

A total of 721 study participants, with 351 participants from the Patioba village and 370 from the Colônia Miranda village, were screened using the KK at baseline, which revealed a positivity rate for *S. mansoni* infection of 33.6 % (118 infected) and 25.7 % (95 infected), respectively. Of the 213 positive participants, one passed away, seven had contraindications for PZQ, and six migrated from their village, and therefore, were not included in the efficacy study of PZQ. Thus, 199 eligible participants were assigned to one of the three treatment groups, maintaining a balanced distribution in terms of sex, age, and parasitic load. Initially, Group 1 consisted of 60 participants, Group 2 of 83 participants, and Group 3 of 56 participants. A total of 93 participants did not submit samples at all follow-up time points and were considered lost to follow-up. For another 18 participants *Schistosoma* positivity could not be confirmed by UCP-LF CAA and were therefore excluded. In total, 88 participants (Group 1 N = 16, Group 2 N = 46, Group 3 N = 26) were included in the final analysis. The flowchart, including recruitment, participant screening, and group formation for the study, is presented in Fig. 2.

**Fig. 3:**
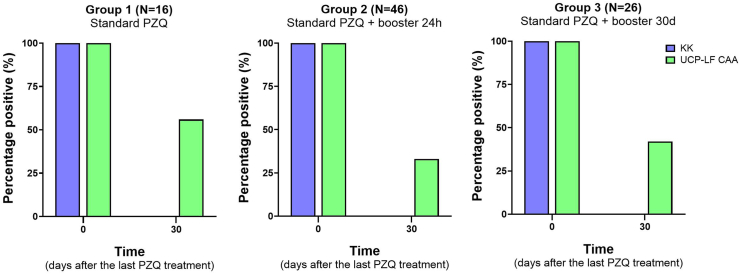
Percentage positive over time in Group 1 (standard dose of PZQ), Group 2 (standard dose of PZQ + booster after 24 h) and Group 3 (standard dose of PZQ + booster after 30 days) based on Kato-Katz (KK, blue) and up-converting reporter particle, lateral flow, circulating anodic antigen (UCP-LF CAA, green) test.

Baseline characteristics of the groups are presented in Table 1. The female gender accounted for 53.4 % (47/88) of the study participants. The mean ages for Group 1, Group 2, and Group 3 were 39 (SD ± 20.1), 35 (SD ± 19.0), and 35 (SD ± 19.9) years, respectively. In all groups, the majority of participants had a low parasite load (>78 %). The percentage positive for *S. mansoni* infection in each group before and after treatment based on KK and UCP-LF CAA is shown in Fig. 3. Other characteristics as well as data on the efficacy estimates based on KK and UCP-LF CAA in each group are presented in Table 1.

**Table 1:** Baseline characteristics of the three different treatment groups and efficacy outcomes of the three different PZQ treatment regimens based on Kato-Katz (KK) and up-converting reporter particle, lateral flow circulating anodic assay (UCP-LF CAA).

	Group 1 Standard PZQ treatment (N = 16)	Group 2 Standard PZQ treatment + booster after 24h (N = 46)	Group 3 Standard PZQ treatment + booster after 30 days (N = 26)
**Age, years**	39.4 ± 20.1	35.0 ± 19.0	35.2 ± 19.9
**Gender**			
Male	7 (43.8 %)	23 (50.0 %)	11 (42.3 %)
Female	9 (56.3 %)	23 (50.0 %)	15 (57.7 %)
**Village**			
Patioba	9 (56.3 %)	31 (67.4 %)	20 (76.9 %)
Colônia Miranda	7 (43.8 %)	15 (32.6 %)	6 (23.1 %)
**Kato-Katz**			
Positive at baseline	16 (100 %)	46 (100 %)	26 (100 %)
Positive at follow up^a^	0	0	0
Cure rate (CR)^b^	100 %	100 %	100 %
Intensity of infection			
Light (1–99 EPG)	13 (81.3 %)	36 (78.3 %)	21 (80.8 %)
Moderate (110–399 EPG)	2 (12.5 %)	9 (19.6 %)	5 (19.2 %)
Heavy (≥400 EPG)	1 (6.3 %)	1 (2.2 %)	0 (0 %)
Median EPG^c^			
Baseline	18	18	33
Follow-up^a^	0	0	0
Arithmetic mean EPG			
Baseline	83.0	56.6	63.0
Follow-up^a^	0	0	0
Egg reduction rate (ERR)^b^	100 %	100 %	100 %
**UCP-LF CAA**			
Positive at baseline	16 (100 %)	46 (100 %)	26 (100 %)
Positive at follow up^a^	9 (56.2 %)	12 (30.4 %)	11 (42.3 %)
Cure rate (CR)^b^	43.8 %	69.6 %	57.7 %
Intensity of infection			
Low (0.6–9 pg/ml)	4 (25.0 %)	14 (30.4 %)	7 (26.9 %)
Moderate (10–99 pg/ml)	9 (56.3 %)	24 (52.2 %)	13 (50.0 %)
High (>100 pg/ml)	3 (18.8 %)	8 (17.4 %)	6 (23.1 %)
Median CAA-level (pg/ml)^c^			
Baseline	22.4	29.3	51.5
Follow-up^a^	1.3	1.4	1.5
Arithmetic mean CAA-level (pg/ml)			
Baseline	113.0	99.3	102.8
Follow-up^a^	2.8	1.3	1.2
Intensity reduction rate (IRR)^b^	97.5 %	98.7 %	98.8 %

Abbreviations: CAA, circulating anodic antigen; EPG, eggs per gram of stool.

a Follow-up was defined as 30 days after the last treatment (i.e. D30 for Group 1 and 2, and D60 for Group 3, see Materials and Methods).

b Cure Rate and Egg/Intensity Reduction Rate estimated 30 days after the last treatment (i.e. D30 for Group 1 and 2, and D60 for Group 3, see Materials and Methods).

c Median of the positives.

According to the KK method, all treatment regimens showed a CR of 100 % (Table 1). However, the UCP-LF CAA test revealed a lower antigenic CR and more evident differences between the treatment regimens: Group 2 showed the highest CR (69.6 %), followed by Group 3 (57.7 %) and lastly Group 1 (43.8 %). Additionally, the CR in Group 1 and Group 2 further increased to 56.3 % and 80.4 % 60 days after treatment, respectively (Supplementary Table 1).

**Fig. 4:**
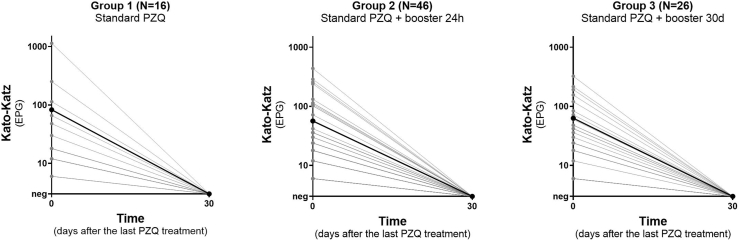
Individual intensity of infection over time based on Kato-Katz in Group 1 (standard dose of PZQ), Group 2 (standard dose of PZQ + booster after 24 h) and Group 3 (standard dose of PZQ + booster after 30 days). Arithmetic mean EPG is indicated by the solid black line.

**Fig. 5:**
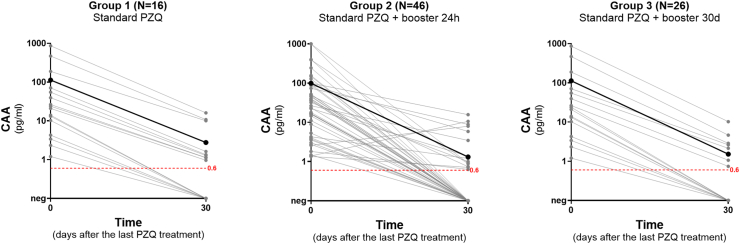
Individual intensity of infection over time based on up-converting reporter particle, lateral flow circulating anodic antigen (UCP-LF CAA) test in Group 1 (standard dose of PZQ), Group 2 (standard dose of PZQ + booster after 24 h) and Group 3 (standard dose of PZQ + booster after 30 days). Arithmetic mean CAA-level is indicated by the solid black line.

The infection intensity by *S. mansoni*, as well as the impact of the different PZQ treatment regimens on this parameter over time, are shown in Table 1, Fig. 4 (based on KK), and Fig. 5 (based on UCP-LF CAA). According to the KK method, most participants presented with low parasite burden at baseline, with an overall arithmetic mean of 65 EPG. An ERR of 100 % was observed in all study groups, treated either with single or repeated PZQ doses. Based on the UCP-LF CAA test, most participants in the three groups presented with low CAA-levels at baseline, which in all groups decreased significantly after treatment. The observed IRR was 97.5 % in Group 1, 98.7 % in Group 2 and 98.8 % in Group 3. The IRR did not significantly increase when measured 60 days after treatment for Group 1 (98.7 %) and Group 2 (99.0 %) (Supplementary Table 1).

Reported adverse events within 24 h after PZQ treatment are shown in Table 2. Dizziness was the most reported symptom after the first dose (all groups) as well as after the second dose (Group 2 and Group 3). The second most reported symptom was vomiting (25.0 %) in Group 1, headache (28.3 %) in Group 2, and nausea (34.6 %) in Group 3. The occurrence of more than one adverse event after the 1st or 2nd doses of PZQ was reported in at least 50 %, 63.0 % vs. 32.6 %, and 61.5 % vs. 38.5 % in Group 1, Group 2, and Group 3, respectively. Overall, repeating PZQ treatment did not result in an increase in the number of adverse events.

**Table 2:** Adverse events reported by the different study groups 24 h after praziquantel treatment.

Type of AEs	Group 1 Standard PZQ treatment (N = 16)		Group 2 Standard PZQ treatment + booster after 24h (N = 46)		Group 3 Standard PZQ treatment + booster after 30 days (N = 26)	
	1° Dose	Repeat dose	1° Dose	Repeat dose	1° Dose	Repeat dose
Dizziness	7 (43.8 %)	–	24 (52.2 %)	13 (28.3 %)	15 (57.7 %)	11 (42.3 %)
Vomiting	4 (25.0 %)	–	11 (23.9 %)	6 (13.0 %)	7 (26.9 %)	8 (30.8 %)
Diarrhea	2 (12.5 %)	–	10 (21.7 %)	4 (8.7 %)	5 (19.2 %)	4 (15.4 %)
Drowsiness	1 (6.3 %)	–	11 (23.9 %)	6 (13.0 %)	2 (7.7 %)	3 (11.5 %)
Nausea	3 (18.8 %)	–	12 (26.1 %)	6 (13.0 %)	9 (34.6 %)	4 (15.4 %)
Headache	3 (18.8 %)	–	13 (28.3 %)	5 (10.9 %)	5 (19.2 %)	3 (11.5 %)
Urticaria	–	–	1 (2.2 %)	–	–	–
Malaise	3 (18.8 %)	–	11 (23.9 %)	5 (10.9 %)	4 (15.4 %)	2 (7.7 %)
Fever	–	–	1 (2.2 %)	–	–	–
Other symptoms	1 (6.3 %)	–	–	3 (6.5 %)	1 (3.8 %)	4 (15.4 %)
Reported occurrence of more than one adverse event	8 (50.0 %)	–	29 (63.0 %)	15 (32.6 %)	16 (61.5 %)	10 (38.5 %)
Did not report the occurrence of an adverse event	4 (25.0 %)	–	7 (15.2 %)	20 (43.5 %)	4 (15.4 %)	7 (26.9 %)

## 4 Discussion

This study was conducted in the context of schistosomiasis treatment in Brazil, where we evaluated the effectiveness of different treatment regimens with PZQ, based on single versus spaced double dosing, using *Schistosoma mansoni* eggs and *Schistosoma* Circulating Anodic Antigen (CAA) as outcomes. Based on KK, all treatment regimens showed a CR and ERR of 100 %. Using more sensitive CAA detection, different patterns were observed: although intensity reduction rates were high across all treatment groups (>97 %), the CR significantly improved with repeated treatment compared to the single-dose control group. The 24 h booster achieved the highest CR (69.6 %, Group 2), followed by the 30-day booster (57.7 %, Group 3), both outperforming the single-dose treatment (43.8 %, Group 1). This indicates that while the reduction in infection intensity was similar between groups, repeating treatment at a short interval is more effective at achieving complete clearance of infection. Repeating PZQ treatment did not result in an increase in the number of adverse events and all observed adverse events were mild and transient.

The observed variation in PZQ efficacy between egg loads and circulating antigen detection methods is consistent with previous findings, from African settings (Coulibaly et al., 2013; Sousa et al., 2019; Hoekstra et al., 2020; Mazigo et al., 2021; Hoekstra et al., 2022a) as well as in a study from Brazil (Sousa et al., 2019). The therapeutic limitations of PZQ against juvenile *Schistosoma* stages (Doenhoff et al., 2008) may impact treatment efficacy in endemic settings where individuals may harbor different stages of the parasite (Ally et al., 2024). Since immature parasites are still developing into adults at the time of evaluation and do not yet produce eggs, egg-based efficacy estimates may be underestimated. Additionally, although PZQ is effective against adult *Schistosoma*, also some of these forms may escape its action (Botros et al., 2005; Pica-Mattoccia and Cioli, 2004), leading not to complete elimination of the worms but rather to a reduction in their population (Colley et al., 2017), reflected by detectable CAA-levels after treatment. Changes in parasite fertility, including reduced oviposition in adult worms that survive PZQ treatment, have also been reported (Winkelmann et al., 2021; Nogueira et al., 2022). The resulting reduction in host parasite load may lead to false-negative results for infection when using KK, due to its low sensitivity in detecting light infections (Bärenbold et al., 2017), leading to overestimated CRs and ERRs.

Considering the parasitic load of *S. mansoni,* the efficacy of PZQ was deemed satisfactory, regardless of whether it was administered in a single or double dose, as well as the diagnostic method used. The ERR (based on KK) for the different treatment regimens against *S. mansoni* infection was 100 %, and the IRR obtained through UCP-LF CAA also reached the limit set by the WHO (≥90 %) (WHO, 2013b), which aligns with the outcomes of a recent systematic review concluding that, although PZQ does not completely clear the infection in some individuals, it significantly reduces the intensity of the infection (Fukushige et al., 2021). The WHO recommends that the evaluation of the efficacy of PZQ be based on the reduction in intensity of infection (e.g. ERR) and not just on the CR (WHO, 2013b). Despite the marked reduction in infection intensity observed in the egg counts and CAA-levels, reflecting a reduction in parasitic load, the failure to eliminate the infection in some individuals, as evidenced by lower CRs based on UCP-LF CAA, is a concerning factor. To interrupt the transmission of schistosomiasis and advance towards elimination, it is important that treatment is effective in achieving complete cure in all infected individuals and results from this study show that repeated treatment should be considered to increase clearance of infection. Also, the time point for estimating efficacy of treatment should be further investigated, as CAA-levels decreased further over time in Group 1 and Group 2 leading to higher CRs in these groups (see Supplementary Fig. 1 and Supplementary Table 1).

Factors such as the burden of *Schistosoma* infection and the characteristics of the analyzed population may be correlated with the effectiveness of the treatment. The results from our study revealed a moderate prevalence of schistosomiasis in both villages (Patioba and Colônia Miranda). Furthermore, approximately 75 % of the positive individuals reported having contact with the local natural waters. Activities related to agriculture, subsistence, fishing, as well as domestic activities, personal hygiene, and leisure, are the main reasons for contact with these waters. Water supply and sanitation infrastructure in both villages are inadequate or nonexistent, and the disposal of solid and liquid waste is done in open areas and/or in rudimentary pits. The presence of *Biomphalaria* snails on the streets is prominent (personal observations), and the lack of drainage and management of rainwater exposes residents to the risk of repeated *S. mansoni* infections even within the peridomestic area. In such scenarios, individuals are frequently exposed and most likely host various stages of the parasite, so repeated PZQ treatment would target both mature and immature forms, potentially leading to better cure. However, treatment does not prevent the imminent risk of reinfection, which remains a challenge to overcome (King et al., 2011).

All treatment regimens with PZQ in this study were well tolerated by the participants, with all observed adverse events being mild and transient, and dizziness being the most frequently reported by all groups. In groups 2 and 3, a higher proportion of adverse events occurred after the first dose, and a decline in this frequency was observed after the second dose of PZQ. Our findings are similar to the adverse events found in other studies, where treatment with PZQ was administered at a dose of 60 mg/kg in repeated doses (da Cunha et al., 1987) or in a single dose (Olliaro et al., 2011). The high intensity of infection is a predictor of a higher incidence of adverse events associated with treatment (Mnkugwe et al., 2021), and the mild and transient events reported in this study may be a result of the *S. mansoni* load in the participants being predominantly low. Additionally, the snack provided to participants before treatment may also have contributed to the reduction of adverse events (Muhumuza et al., 2014; Nalugwa et al., 2015). Pharmacogenetic factors also seem to influence the incidence and intensity of adverse events related to PZQ, as previously reported in African populations carrying certain defective variant alleles (Mnkugwe et al., 2021). This may also explain the difference between the main report of events in this study and previous studies conducted on the African continent, where abdominal/stomach pain/discomfort were the most frequently reported adverse events, particularly at a dosage of 40 mg/kg of PZQ (Coulibaly et al., 2012; Nalugwa et al., 2015; Hoekstra et al., 2020; Tadele et al., 2023; N'Goran et al., 2023). It is worth noting that the African studies assessing efficacy are conducted with a lower dosage (40 mg/kg) of PZQ and in preschool/school-aged children, where inconsistencies in safety assessment methodologies for this target population may underestimate reports of adverse events related to PZQ (Zwang and Olliaro, 2017).

One limitation of this study is the group allocation procedure: while it was intended to balance sex, age and infection intensity (based on KK), no formal randomization procedure was followed leading to a potential risk of selection bias. Furthermore, the relatively high proportion of participants lost to follow-up – primarily due to occupational absences at scheduled community follow-up visits – may have introduced attrition bias, potentially influencing the interpretability and generalizability of our findings. Although a larger proportion of participants was initially assigned to Group 2 due to concerns about potential losses from the short interval between the two treatment doses and the risk of refusing a second treatment due to potential adverse events, the number of participants lost to follow-up was similar across all three groups. This suggests that loss to follow-up occurred largely at random and was unlikely to be related to treatment-related adverse events, reducing the likelihood that attrition introduced bias in our study outcomes. Our study showed a potential difference in treatment response between the three groups, which should be further evaluated in a randomized controlled trial. Discrepancies between KK and UCP-LF CAA led to the exclusion of 18 participants from the current analysis. The absence of CAA despite the presence of eggs may have been caused by low infection intensity (17 of the 18 cases were light intensity infections, 1–99 EPG), although sample processing errors cannot be excluded either. Conversely, in a random sub-selection of 82 individuals who were negative by KK, the more sensitive UCP-LF CAA test identified an additional 26 positive cases (Supplementary Fig. 2). Unfortunately, due to budget as well as practical constraints, it was not possible to perform the UCP-LF CAA test on all samples collected at baseline. Nevertheless, a good overall correlation between egg loads and CAA concentrations was observed at baseline (Supplementary Fig. 2). While KK remains valuable for detecting moderate-to-heavy intensity infections, the inclusion of antigen detection provides a more comprehensive picture of infection dynamics. The detection of additional CAA-positive cases among KK-negative individuals suggests that relying only on microscopy would underestimate the true infection prevalence, resulting in under-treatment and overestimation of treatment efficacy.

In conclusion, this study demonstrated that repeated PZQ treatment was more effective against *S. mansoni* infection, achieving the highest antigen-based CR compared to the single-dose regimen. All treatment regimens were safe, with only mild and transient adverse events reported. Reduction in intensity of infection, based on both egg counts (ERR) and CAA-levels (IRR), was within the performance targets recommended by the WHO across all treatment regimens. Nevertheless, despite the improvement in CR following repeated PZQ treatment, the antigen-based CR was sub-optimal. Achieving effective control and eventual interruption of transmission will require combining efficacious treatment strategies with sensitive diagnostic tools that accurately detect active infections, alongside targeted measures addressing local transmission dynamics and risk factors in endemic communities.

## CRediT authorship contribution statement

**Rosangela Lima de Freitas Galvão:** Writing – review & editing, Writing – original draft, Visualization, Validation, Methodology, Investigation, Formal analysis, Conceptualization. **Pytsje T. Hoekstra:** Writing – review & editing, Writing – original draft, Validation, Supervision, Software, Resources, Methodology, Investigation, Funding acquisition, Formal analysis, Conceptualization. **Paul L.A.M. Corstjens:** Writing – review & editing, Resources, Methodology, Funding acquisition, Conceptualization. **Marta Cristhiany Cunha Pinheiro:** Writing – review & editing, Validation, Methodology, Investigation, Data curation, Conceptualization. **Angela Maria da Silva:** Visualization, Validation, Resources, Methodology, Investigation. **Luciene Barbosa:** Visualization, Resources, Methodology, Investigation. **Sidney Lourdes César Souza Sá:** Writing – review & editing, Validation, Resources, Methodology, Investigation. **Govert J. van Dam:** Writing – review & editing, Resources, Methodology, Funding acquisition, Conceptualization. **Fernando Schemelzer de Moraes Bezerra:** Writing – review & editing, Visualization, Validation, Supervision, Resources, Project administration, Methodology, Investigation, Funding acquisition, Data curation, Conceptualization.

## Funding

This work was funded by the Council for Scientific and Technological Development (10.13039/501100003593CNPq)/Ministry of Science, Technology and Innovations/10.13039/100015539Federal Government of Brazil (Process No. 404966/2021-7/10.13039/501100003593CNPq/MCTI/FNDCT N° 18/2021); by the Ceará Foundation for Scientific and Technological Development Support (10.13039/501100005283FUNCAP) (Process No. BMD-0008-01273.01.01/22).

## Conflict of interest statement

The authors declare that there is no conflict of interest related to this research.
