# Supplementary material for: Evaluating the efficacy of different praziquantel treatment regimens using egg and circulating anodic antigen (CAA) detection methods in a Schistosoma mansoni endemic area in northeastern Brazil
Source: Int J Parasitol Drugs Drug Resist. 2025 Nov 26;29:100628. doi: 10.1016/j.ijpddr.2025.100628 (PMC12719093; doi:10.1016/j.ijpddr.2025.100628)
Supplement: Multimedia component 1 [file mmc1.docx]

**Supplementary files.**

**Supplementary Table 1.** Baseline characteristics of treatment groups and efficacy outcomes of PZQ through the Kato-Katz (KK) and Up-Converting reporter Particle, lateral flow circulating anodic assay (UCP-LF CAA) at different follow-up times.

|  | **Group 1**  (N=16) | **Group 2**  (N=46) | **Group 3**  (N=26) |  |
| --- | --- | --- | --- | --- |
| **Age, years** | 39.4 ± 20.1 | 35.0 ± 19.0 | 35.2 ± 19.9 |  |
| **Gender** |  |  |  |  |
| Male | 7 (43.8%) | 23 (50.0%) | 11 (42.3%) |  |
| Female | 9 (56.3%) | 23 (50.0%) | 15 (57.7%) |  |
| **Village** |  |  |  |  |
| Patioba | 9 (56.3%) | 31 (67.4%) | 20 (76.9%) |  |
| Colônia Miranda | 7 (43.8%) | 15 (32.6%) | 6 (23.1%) |  |
| **Kato-Katz** |  |  |  |  |
| Positive at baseline | 16 (100%) | 46 (100%) | 26 (100%) |  |
| Negative at follow up (D30) | 16 (100%) | 46 (100%) | 25 (96.2%) |  |
| Negative at follow up (D60) | 16 (100%) | 46 (100%) | 26 (100%) |  |
| Cure rate (CR)* | 100% | 100% | 100% |  |
| Cure rate (CR) (D60)** | 100% | 100% |  |  |
| Intensity of infection |  |  |  |  |
| Light (1-99 EPG) | 13 (81.3%) | 36 (78.3%) | 21 (80.8%) |  |
| Moderate (110-399 EPG) | 2 (12.5%) | 9 (19.6%) | 5 (19.2%) |  |
| Heavy (≥400 EPG) | 1 (6.3%) | 1 (2.2%) | 0 (0%) |  |
| Median EPG*** |  |  |  |  |
| Baseline | 18 | 18 | 33 |  |
| D30 | 0 | 0 | 0 |  |
| D60 | 0 | 0 | 0 |  |
| Arithmetic mean EPG |  |  |  |  |
| Baseline | 83.0 | 56.6 | 63 |  |
| D30 | 0 | 0 | 0.5 |  |
| D60 | 0 | 0 | 0 |  |
| Egg reduction rate (ERR)* | 100% | 100% | 100% |  |
| Egg reduction rate (ERR) (D60)** | | 100% | 100% |  |
| **UCP-LF CAA** |  |  |  |  |
| Positive at baseline | 16 (100%) | 46 (100%) | 26 (100%) |  |
| Negative at follow up (D30) | 7 (43.8%) | 34 (69.6%) | 13 (50.0%) |  |
| Negative at follow up (D60) | 9 (56.3%) | 37 (80.4%) | 15 (57.7%) |  |
| Cure rate (CR)* | 43.8% | 69.6% | 57.7% |  |
| Cure rate (CR) (D60)** | 56.3% | 80.4% |  |  |
| Intensity of infection |  |  |  |  |
| Low (0.6-9 pg/ml) | 4 (25.0%) | 14 (30.4%) | 7 (26.9%) |  |
| Moderate (10-99 pg/ml) | 9 (56.3%) | 24 (52.2%) | 13 (50.0%) |  |
| High (>100 pg/ml) | 3 (18.8%) | 8 (17.4%) | 6 (23.1%) |  |
| Median CAA-level (pg/ml)*** |  |  |  |  |
| Baseline | 22.4 | 29.3 | 51.5 |  |
| D30 | 1.3 | 1.4 | 2.0 |  |
| D60 | 2.6 | 1.2 | 1.5 |  |
| Arithmetic mean CAA-level (pg/ml) |  |  |  |  |
| Baseline | 113.0 | 99.3 | 102.8 |  |
| D30 | 2.8 | 1.3 | 1.8 |  |
| D60 | 1.5 | 0.3 | 1.2 |  |
| Intensity reduction rate (IRR)* | 97.5% | 98.7% | 98.8% |  |
| Intensity reduction rate (IRR) (D60)** | 98.7% | 99.0% |  |  |

*Cure rate and Egg/Intensity Reduction Rate estimated 30 days after the last treatment (i.e. D30 for Group 1 and Group 2 and D60 for Group 3).

** Cure rate and Egg/Intensity Reduction Rate estimated 60 days after the last treatment for Group 1 and Group 2.

***Median of the positives.

Abbreviations: EPG, eggs per gram of stool; SD, Standard deviation; D30, day 30; D60, day 60.


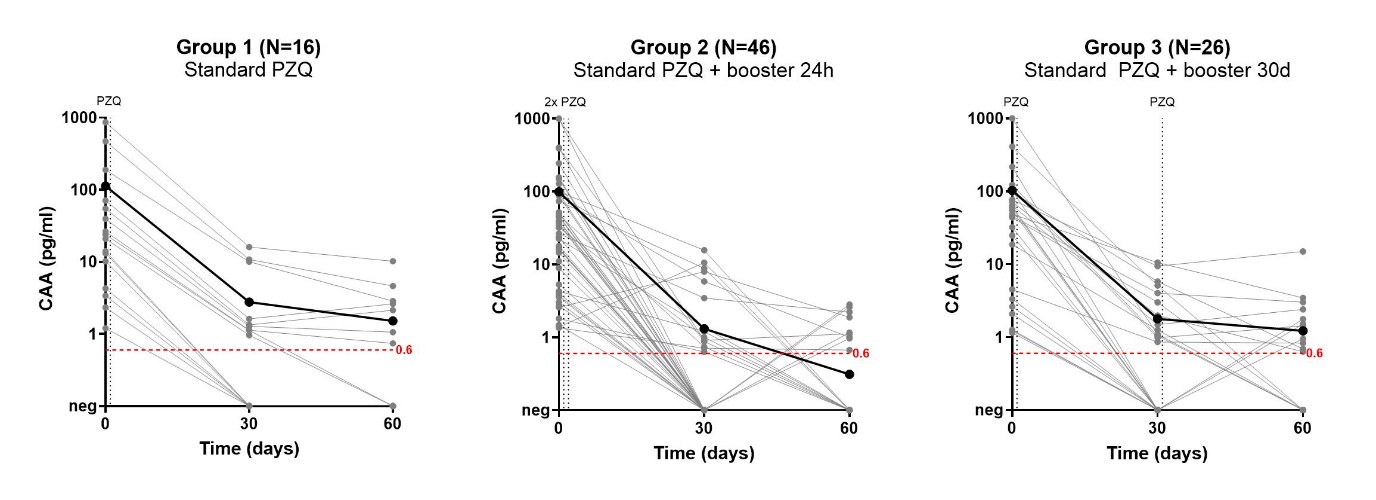


**Supplementary Figure 1**. Individual intensity of infection over time based on up-converting reporter particle, lateral flow circulating anodic antigen (UCP-LF CAA) test in Group 1 (standard dose of PZQ), Group 2 (standard dose of PZQ + booster after 24 hours) and Group 3 (standard dose of PZQ + booster after 30 days).

**
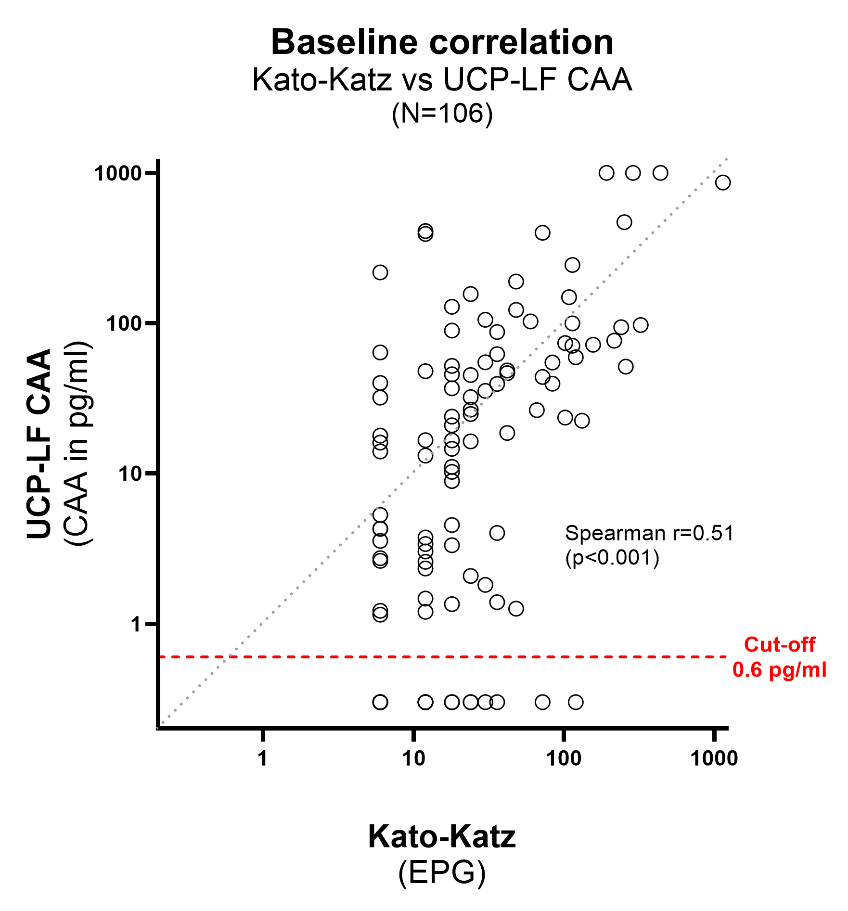
**

**Supplementary Figure 2**.  Baseline correlation between Kato-Katz and UCP-LF CAA.
